# A peptide fragment from the human COX3 protein disrupts association of *Mycobacterium tuberculosis* virulence proteins ESAT-6 and CFP10, inhibits mycobacterial growth and mounts protective immune response

**DOI:** 10.1186/1471-2334-14-355

**Published:** 2014-07-01

**Authors:** Sachin Kumar Samuchiwal, Sultan Tousif, Dhiraj Kumar Singh, Arun Kumar, Anamika Ghosh, Kuhulika Bhalla, Prem Prakash, Sushil Kumar, Maitree Bhattacharyya, Prashini Moodley, Gobardhan Das, Anand Ranganathan

**Affiliations:** 1 Recombinant Gene Products Group, International Centre for Genetic Engineering and Biotechnology, ICGEB, Aruna Asaf Ali Marg, New Delhi 110067, India; 2 Immunology Group, International Centre for Genetic Engineering and Biotechnology, ICGEB, Aruna Asaf Ali Marg, New Delhi 110067, India; 3 Department of Biochemistry, University of Calcutta, 35, Ballygunge Circular Road, Kolkata 700 019, India; 4 School of Laboratory Medicine, College of Health Sciences, University of Kwazulu-Natal, Durban, South Africa

**Keywords:** Tuberculosis, Human COX3, ESAT-6, CFP10, Protein-protein interactions, Th1, Th17

## Abstract

**Background:**

Tuberculosis (TB) is one of the most prevalent infectious diseases affecting millions worldwide. The currently available anti-TB drugs and vaccines have proved insufficient to contain this scourge, necessitating an urgent need for identification of novel drug targets and therapeutic strategies. The disruption of crucial protein-protein interactions, especially those that are responsible for virulence in *Mycobacterium tuberculosis* – for example the ESAT-6:CFP10 complex – are a worthy pursuit in this direction.

**Methods:**

We therefore sought to improvise a method to attenuate *M. tuberculosis* while retaining the latter’s antigenic properties. We screened peptide libraries for potent ESAT-6 binders capable of dissociating CFP10 from ESAT-6. We assessed the disruption by a peptide named HCL2, of the ESAT-6:CFP10 complex and studied its effects on mycobacterial survival and virulence.

**Results:**

We found that HCL2, derived from the human cytochrome c oxidase subunit 3 (COX3) protein, disrupts ESAT-6:CFP10 complex, binds ESAT-6 potently, disintegrates bacterial cell wall and inhibits extracellular as well as intracellular mycobacterial growth. In addition, an HCL2 expressing *M. tuberculosis* strain induces both Th1 and Th17 host protective responses.

**Conclusions:**

Disruption of ESAT-6:CFP10 association could, therefore, be an alternate method for attenuating *M. tuberculosis*, and a possible route towards future vaccine generation.

## Background

Tuberculosis (TB) remains the leading cause of infectious disease-related deaths worldwide. There are an estimated 8.5-9.2 million cases and 1.2-1.5 million deaths caused by *Mycobacterium tuberculosis (M.tb)* each year [1]. One-third of the global population is infected with the latent form of TB, a scenario translating into an enormous reservoir for a future pandemic. BCG, the only available vaccine, provides significant protection against disseminated and tubercular meningitis, but has very little or no efficacy against adult pulmonary TB [2]. We have earlier shown that an optimum host protection against TB requires both Th1 and Th17 responses [3]. BCG induces Th1 response, but fails to induce Th17 response in the lung, a factor mostly responsible for the inferior efficacy of BCG [3,4]. Considerable efforts have been made to generate recombinant BCG but with little headway [5,6]. On the other hand, the currently available anti-TB chemotherapy, DOTS, is lengthy, consists of multiple antibiotics that cause severe hepatotoxicity and is effective in only 50% of MDR-TB cases [7,8]. Therefore, identification of novel therapeutic strategies is urgently needed.

A promising approach can be the targeting of important protein-protein interactions within the pathogen, with an emphasis on those proteins that are the source of mycobacterial virulence. As of now, there exist no peptides or small molecules that specifically target bacterial virulence factors. Such moieties can conveniently be used along with conventional drugs to improve TB chemotherapy and in combating multi-drug resistance [9]. Quite often virulence is dependent on protein-protein interactions, an area that is as yet unexplored for the development of advanced therapeutics. Recently, it has been shown that ESAT-6, a protein derived from the region of difference (RD-1), plays a critical role in mounting Th17 responses in the lung [3,4]. As stated earlier, we showed that the vaccine strain of BCG lacks the RD-1 region and is therefore unable to generate Th17 responses. Similarly, the RD-1 mutant of *H37Rv* also fails to induce Th17 responses [3]. Although RD-1 recombinant BCG generates both Th1 and Th17 responses, this strain is unsuitable as a vaccine because RD-1 addition makes BCG virulent [10]. It was, therefore, our aim to elucidate the role of interaction between RD-1 components and their potential therapeutic interventions.

In *M.tb*, ESAT-6 and CFP10 proteins are encoded by the RD1 region (9.5 kb in length comprising 9 genes – part of ESX1 secretion system), and secreted as a tight 1:1 heterodimer with a dissociation constant of 1.1 × 10^-8^ M [11,12]. It is well known that the ESAT-6:CFP10 complex plays pivotal role in the pathogenesis of TB. Indeed, deletion of ESAT-6 or RD1 locus attenuates *M.tb* and dramatically reduces vaccine efficacy of the attenuated mutants [3,13]. The many crucial roles of ESAT-6 include membrane lytic activity due to its helix-turn-helix structure and hydrophobic nature that helps mycobacteria escape from phagolysosome and spread [14], as well as macrophagial aggregation and granuloma formation, leading to intercellular bacterial dissemination [15]. It is becoming increasingly clear that ESAT-6 is decisive for the virulence, pathogenesis, and survival of *M.tb*.

Therefore, to understand whether *in vivo* disruption of a protein complex that involves ESAT-6 as a primary component has an effect on the pathogenesis of *M. tuberculosis,* we screened peptides that bind to ESAT-6 with potent affinity and are capable of dissociating CFP10 from ESAT-6. We report here the strong binding capability of a peptide named HCL2 (amino acid sequence: ESTYQGH-HTPPVQKGLRYGIILFITSEVFFFAGFF*), a fragment from human mitochondrial protein COX3, with ESAT-6. We assessed the disruption by HCL2 of the ESAT-6:CFP10 complex by employing a Bacterial Three-Hybrid system recently developed in our laboratory [16] and report a direct correlation in HCL2 expression and ESAT-6:CFP10 disruption. We determined the effects of exogenous and endogenous HCL2 on the growth of *M.tb* and observed significant bacteriocidal effects and changes in cellular and colony morphology. Furthermore, we report the generation of an HCL2-expressing *H37Rv* strain, infection by which induces IL-12 as well as IL-6 production in macrophages, which are the polarizing cytokines for Th1 and Th17 differentiations respectively. Therefore the present study extends our earlier studies regarding BCG and Th17 responses [3], and establishes as a proof of concept, the development of an alternative strain for therapeutic intervention. In addition, HCL2 could also serve as a template for designing new and improved peptide-based therapeutics against TB.

## Methods

### Antibodies and reagents

We used rabbit anti-ESAT-6 antibody (Abcam, Cambridge, MA), mouse anti-His_6X_ antibody (Sigma-Aldrich, St. Louis MA), anti-FLAG antibody (Sigma-Aldrich, St. Louis MA), HRP conjugated anti-mouse IgG (Bio-rad, Hercules, CA), anti-rabbit FITC conjugated secondary antibody (Jackson-ImmunoResearch, West Grove, PA) and anti-mouse Texas Red secondary antibody (Jackson-ImmunoResearch, West Grove, PA) for *in vitro* studies. For *in vivo* studies, anti-CD4 (clone: GK1.5)-FITC, -PerCP-Cy5 or -APC, anti-CD8 (clone: 53–6.7)-FITC, -PerCP-Cy5 or -APC, anti-CD44 (clone: IM7)-APC, anti-Brdu (clone: Bu20a)-PE, anti-CD11b (clone: M1/70)-APC, anti-CD11c (clone: N418)-APC, 7AAD, anti-IFN-γ (clone: XMG1.2)-APC, anti-IL-17 (clone: TC11-18H10.1)-PE, anti-IL-4 (clone: 11B11)-PE, anti-IL-6 (clone: MPS-20 F3)-PE, anti-IL-12 (clone: C15.6)-PE, anti-IL-22 (clone: Poly5164)-PE, anti-IL-10 (clone: JES5-16E3)-PE, anti-IL-9 (clone: MH9A4)-PE, anti-TNF-α (clone: MP6-XT22)-PE, (all from Biolegend, USA) and anti-CD69 (clone: H1.2 F3)-PE (from eBiosciences, USA) were used.

### Mice

BALB/c female mice at 6–8 weeks of age were used throughout this study, following institutional ethical committee guidelines. All animal experiments were conducted in accordance with guidelines approved by the Institutional Animals Ethics Committee of ICGEB, New Delhi, India and Department of Biotechnology (DBT), Government of India. Mice were housed under barrier conditions in a Biosafety Level III laboratory.

### Bacterial two-hybrid studies

Bacteriomatch™ system was used as described earlier [16,17]. Two colonies each from HCL2pTRGnn + ESAT-6pBTnn, positive and negative, were grown in cultures and patched on X-gal indicator plate. Interactions were validated by liquid β-Galactosidase assay and compared with well-established mycobacterial interaction between ESAT-6 and CFP10 (ESAT-6pBTnn + CFP10pTRGnn).

### *In vitro* Far-Western Dot Blot analysis

For Far-Western Dot Blot assay, ESAT-6-FLAG was purified by ion-exchange chromatography. Briefly, *E. coli* BL21 (DE3) cells harboring flagesat6pET28a were subjected to IPTG induction and cells were harvested, lysed in denaturing condition. Clear supernatant was allowed to bind on DEAE-sepharose (Amersham) followed by washing with lysis buffer and the resin bound proteins were eluted using a gradient of NaCl from 0 M to 1 M, prepared in lysis buffer. Purified protein was refolded by sequential dialysis against PBS with decreasing concentration of urea. The interaction between ESAT-6-FLAG and HCL2-His_6X_ (commercially synthesized from GenScript, Hong Kong) was determined using a protocol reported previously [16]. An unrelated protein ED3-His_6X_ (sequence given in Additional file 1: Figure S1) was used as a negative control. CFP10-His_6X_ and ESAT-6-FLAG were used as positive controls.

### Cloning of HCL2 in pMTSA vector

The ORF encoding HCL2 peptide was amplified from original lung cDNA library clone using 5’-AAGGATCCTACGTAAGAATTCGGCACGAG-3’ and 5’-AAGGATCCTACGTAGAAAA ATCCTGCGAAGAAAA-3’ as forward and reverse primers respectively. The gene was cloned in a *Sna*BI site in an arabinose-inducible three-hybrid vector as previously described [16]. Expression of HCL2-His_6X_ was analyzed on Tricine-SDS PAGE and Western Blotting was done using Anti-His_6X_ antibodies.

### Bacterial Three-Hybrid and arabinose gradient liquid β-Galactosidase assay

Bacterial Three-Hybrid studies were carried out as described earlier [16]. To study the correlation between disruption of the ESAT-6:CFP10 interaction and *in vivo* expression levels of HCL2, an Arabinose gradient liquid β-Galactosidase assay was performed. The experiment was carried out in triplicates by varying L-arabinose concentration ranging from 0 to 1%. Western Blotting was done with increasing concentration of L-arabinose (0-1%), to show the gradual increase in HCL2-His_6X_ induction corresponding to decrease in β-Galactosidase activity.

### HCL2 cloning in a mycobacterial shuttle vector

The ORF expressing HCL2 peptide was cloned in mycobacterial constitutive expression vector pVV16 and pVVGFPHis_6X_ and used to electroporate *H37Rv* electro-competent cells, as described earlier [17].

### Effect of HCL2 on *M. tuberculosis* growth

We examined the effects of HCL2 peptide on *M.tb* growth by using two methods. First, by electroporating HCL2pVV16 (*H37Rv/HCL2;* strain expressing HCL2-His_6X_) and secondly by adding HCL2-His_6X_ peptide to the culture exogenously at a final concentration of 15 μg/ml *(H37Rv + HCL2)*. HCL2 was cloned in mycobacterial constitutive expression vector pVV16 as described earlier [17]. Cultures were inoculated in triplicates each for *H37Rv/HCL2*, *H37Rv + HCL2, H37Rv/pVV16* (strain having only plasmid control)*, ΔRD1, H37Rv/GFP, H37Rv + DL1* and *H37Rv* and assessed spectrophotometrically for 18 days at 630 nm. *H37Rv* was added with equivalent amount of Milli-Q water used to dissolve the peptide HCL2-His_6X_. A higher concentration of HCL2-His_6X_ was not used due to precipitation of the peptide at concentrations > 15 μg/ml. Strain lacking RD1 region (*ΔRD1*; kind gift of Prof. David R. Sherman)*, H37Rv* expressing GFP (*H37Rv/GFP*), and addition of an unrelated peptide (sequence given in Additional file 1: Figure S1) DL1 (*H37Rv + DL1*) were used as controls. Another ESAT6 binding peptide, SL3, from our previous studies [16] was also analyzed for its effects on mycobacterial growth during this study (unpublished results).

### Effect of HCL2 on cellular and colony morphology

Electron microscopy was carried out to examine the effect of endogenous or exogenous HCL2 on cellular morphology of *M.tb. H37Rv/HCL2, H37Rv + HCL2, H37Rv, ΔRD1* and *H37Rv/pVV16* cells were fixed by treating with 2% paraformaldehyde solution and adsorbed on a 300 mesh copper grid and air dried. Samples were stained with 1% uranyl acetate followed by photography using a FEI Tecnai 12 Electron Microscope. *H37Rv, H37Rv/pVV16* and *ΔRD1* strains were used as controls. In addition, colony morphology of *H37Rv/HCL2* strain was observed on 7H11 plates and colony texture was compared with *H37Rv*.

### Infection of THP-1 cells by *M. tuberculosis* in presence of HCL2 peptide

Approximately 12,000/well phorbol-12-myristate-13-acetate (Sigma, USA) activated THP-1 cells in a 96 well flat bottom tissue culture plate were infected with *M.tb H37Rv/HCL2* at an MOI of 1:10 using a protocol described previously [18]. Cells were harvested, lysed and plated on 7H11 agar at 0, 24, 48 and 72 hours and CFU counts determined. For effects of exogenous HCL2-His_6X_ (*H37Rv + HCL2*), peptide was added to RPMI media at a final concentration of 15 μg/ml and this media was added to *H37Rv* infected tissue-culture plates at 0 hours and changed after every 24 hours till 72 hours. *H37Rv, H37Rv/GFP, H37Rv + DL1* and *ΔRD1* were used as control strains to infect THP-1 cells. In this experiment, SL3 peptide was also analyzed for its effects on intracellular survival (unpublished results).

### Mice infection with *H37Rv/HCL2*, CFU counts and immunology

BALB/c mice were infected with ~150 CFU of *H37Rv* and *H37Rv/HCL2* using an aerosol chamber. Mice were sacrificed at different time points and cytokine profile and T lymphocytes proliferation were assessed as described earlier [19]. For CFU counts lung and spleen were harvested at different time points and processed as described previously [19].

### Statistical analysis

All experiments were repeated thrice and in triplicates. Mean values were calculated with standard deviation (STDEV) unless stated otherwise. For all statistical analyses Student's T-test was performed to compare two groups; p < 0.05 was considered significant.

## Results

### HCL2 interacts with ESAT-6 *in vitro*

**Figure 1:**
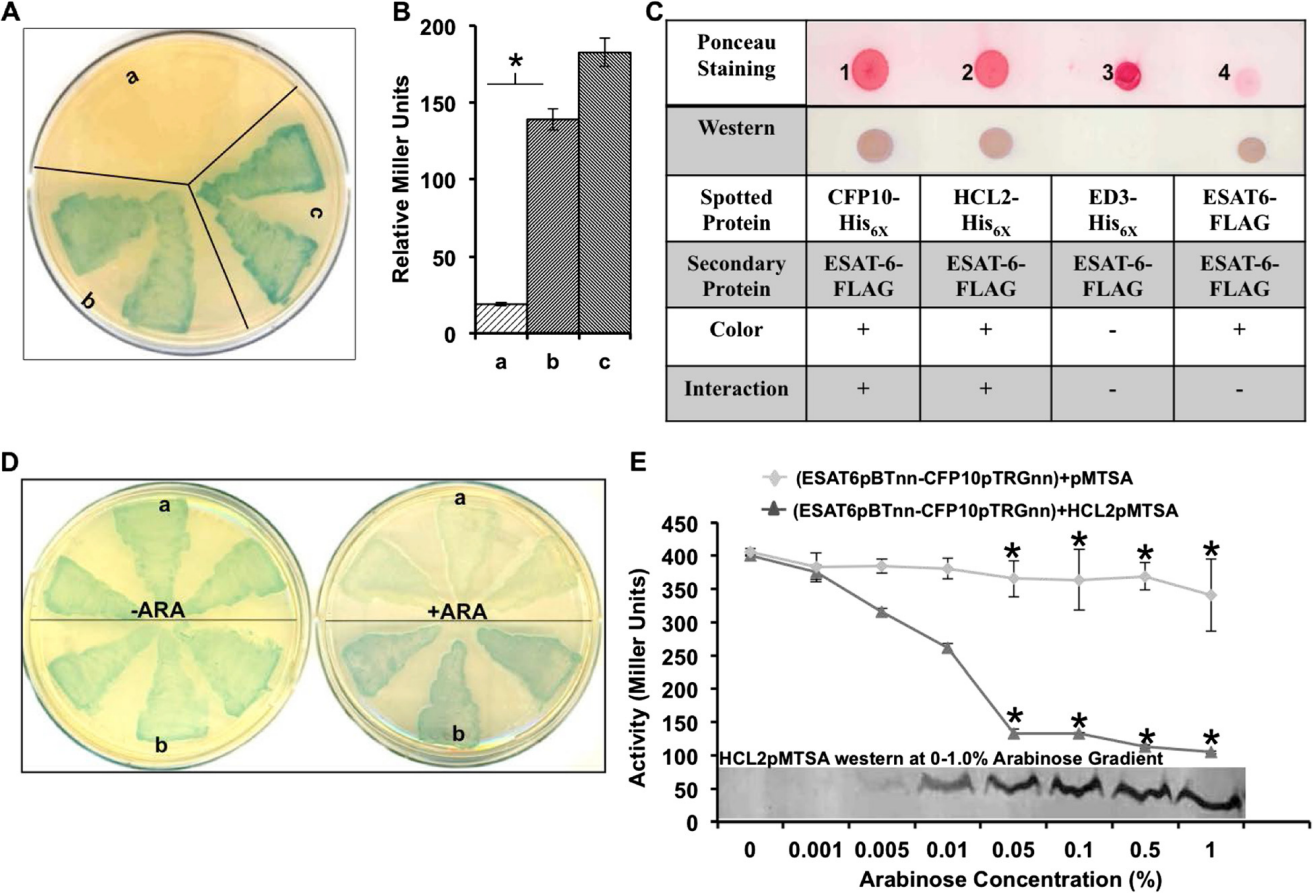
**HCL2 binds with ESAT-6 and disrupts the ESAT-6:CFP10 heterodimeric complex. (A)** Agar plate showing co-transformants: a – ‘white’ negative control pBTnn + ESAT-6pTRGnn; b – interacting clone ESAT-6pBTnn + HCL2pTRGnn; c – ‘blue’ positive control CFP10pTRGnn + ESAT-6pBTnn. **(B)** Interactions between ESAT-6pBTnn + HCL2pTRGnn and ESAT-6pBTnn + CFP10pTRGnn were found comparable by Liquid β-Galactosidase assay. Experiments were repeated thrice and similar observations were made. All readings were found statistically significant by applying Student’s T-test. (*P < 0.05) **(C)** HCL2-His_6X_ interacts with ESAT-6-FLAG *in vitro.* Experiment was repeated thrice and each time positive interaction was observed between HCL2-His_6X_ and ESAT-6-FLAG. **(D)** Disruption of ESAT-6:CFP10 interaction by HCL2 using the three-hybrid system. Three separate colonies from each triple co-transformants plate were picked and patched. a – (ESAT-6pBTnn-CFP10pTRGnn) + HCL2pMTSA; b – (ESAT-6pBTnn-CFP10pTRGnn) + pMTSA. –ARA represents plate without Arabinose induction while + ARA represents plate with Arabinose induction. Experiments were repeated thrice and similar observations were made. **(E)** Bacterial Three-Hybrid results were also corroborated by Arabinose Gradient Liquid β-Galactosidase Assay. Experiment was repeated thrice in triplicates. All readings were found statistically significant by applying Student’s T-test (*P < 0.05).

The genes coding for HCL2 and ESAT-6 were cloned separately in the bacterial two-hybrid vectors pTRGnn and pBTnn, and the vectors used to transform the two-hybrid strain. HCL2pTRGnn + ESAT-6pBTnn (‘+’ indicates presence of both plasmids in the two-hybrid bacterial strain) showed a characteristic blue color comparable to the positive control ESAT-6pBTnn + CFP10pTRGnn, while ESAT-6pTRGnn + pBTnn (negative control) was distinctly white (Figure 1A). Liquid β-Galactosidase assay indicated that HCL2pTRGnn + ESAT-6pBTnn has comparable binding strength to ESAT-6pBTnn + CFP10pTRGnn (Figure 1B). Far-Western Dot Blot revealed positive interaction between ESAT-6-FLAG and HCL2-His_6X_ proteins (Figure 1C). ED3-His_6X_, an unrelated protein, showed no interaction. Loading controls were provided by applying ponceau staining to nitrocellulose membrane just after spotting primary proteins.

### HCL2 can disrupt the ESAT-6:CFP10 heterodimeric complex

Bacterial three-hybrid studies demonstrated that presence of HCL2 peptide in a cell expressing ESAT-6 and CFP10 changes the colony color from blue to white, thereby disrupting ESAT-6:CFP10 interaction (Figure 1D). An arabinose gradient liquid β-Galactosidase assay exhibited a concomitant decrease in β-Galactosidase activity with a proportionate HCL2 expression, observed by Western blotting with Anti-His_6X_ antibodies (Figure 1E), confirming a robust expression of HCL2-His_6X_ peptide in three-hybrid experiments.

### HCL2 inhibits mycobacterial growth in axenic cultures

**Figure 2:**
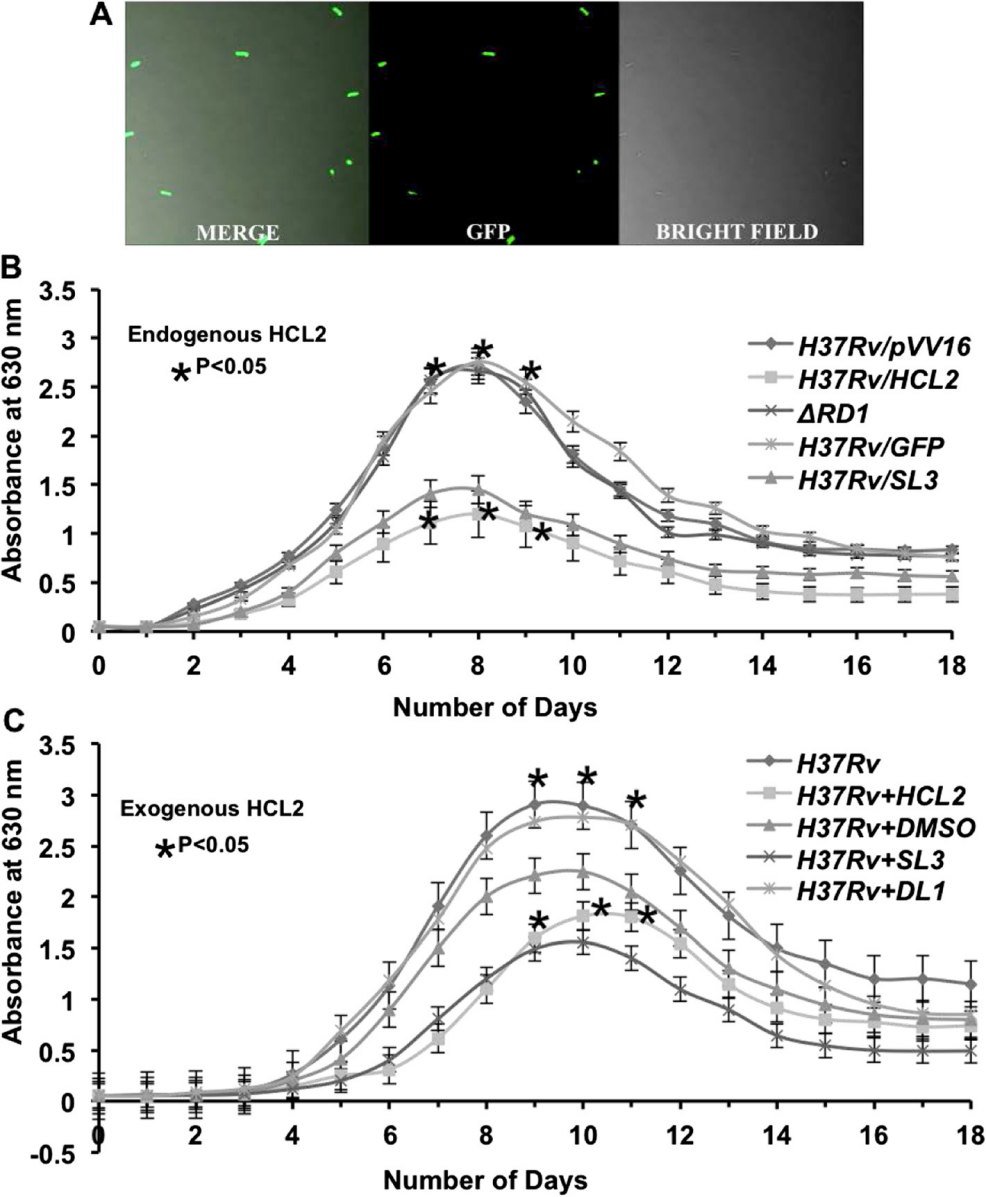
**HCL2 inhibits mycobacterial growth in axenic cultures. (A)** Expression of peptide HCL2-His_6X_ in *H37Rv* cells. Significant reductions were observed in mycobacterial growth in the presence of HCL2 as revealed by growth curves. Reduction with both endogenous **(B)** and exogenous **(C)** HCL2 was found statistically significant by applying Student’s T-test (*P < 0.05).

HCL2 showed significant levels of expression inside *H37Rv* cells as indicated by constitutive expression of HCL2-GFP-His_6X_ in *H37Rv/HCL2GFP* when observed by confocal microscopy (Figure 2A). 91.53% of *H37Rv/HCL2GFP* cells were found to be expressing HCL2 (Additional file 2: Figure S2). We studied mycobacterial growth in liquid cultures and observed dramatically reduced growth in presence of both endogenous as well as exogenous HCL2 in comparison to wild type *H37Rv* and control strains (Figure 2B & 2C).

### HCL2 affects cellular and colony morphology

**Figure 3:**
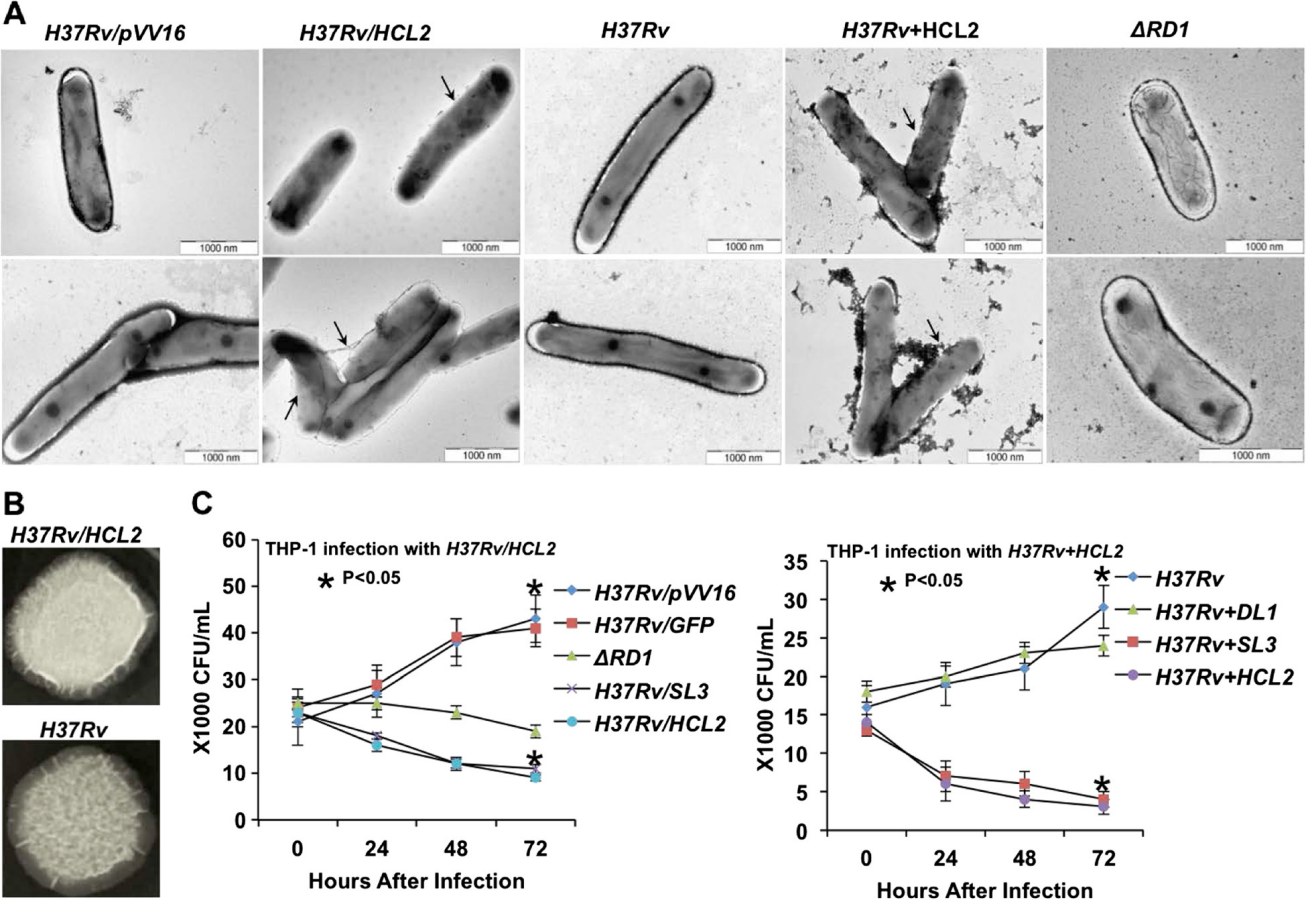
**HCL2 disintegrates bacterial cell wall, changes colony texture and inhibits intracellular growth. (A)** Transmission electron micrographs of mycobacterial cells depicted effects of HCL2 on cell wall integrity and cell shape. **(B)** Representative photographs exhibiting the difference in colony morphology and colony surface texture between *H37Rv* and *H37Rv/HCL2*. **(C)** Significant reductions were found in mycobacterial counts inside THP-1 in the presence of endogenous and exogenous HCL2 peptide. All experiments were conducted in triplicates and repeated at least thrice. (t = 0) defines the time at the beginning of phagocytosis. All readings were found statistically significant by applying Student’s T-test (*P < 0.05).

It is highly likely that accumulation of ESAT-6 within mycobacterial cells may be toxic. To directly observe the effect of HCL2 in *M.tb*, we performed electron microscopy experiments. We found that HCL2 expressing strain is feeble and often possesses a porous membrane (indicated by arrows) (Figure 3A). To further test if exogenously added HCL2 is sufficient to alter *M.tb* cell viability and cell wall damage as observed in HCL2-recombinant *H37Rv*, we treated *M.tb* with HCL2 peptide. Interestingly, we observed similar cell wall disintegration (indicated by arrows) (Figure 3A). Representative images have been provided (Figure 3A), and at least 10 fields were visualized from different grid areas and percentages of cell deformations were calculated (Additional file 3: Figure S3). Furthermore, colony morphology of *H37Rv/HCL2* strain was also observed as appearing smooth when compared to the *H37Rv* (Figure 3B). Colonies from 4 different plates were observed and representative photographs have been provided.

### HCL2 inhibits mycobacterial growth in THP-1 cell line

It is clear that expression of HCL2 in *M. tuberculosis* renders slow growth in liquid cultures. This however does not reflect the status of virulence. Indeed, some of the drug resistant and mutant strains grow slower than the parent *H37Rv*[20]. In an intracellular survival experiment, we observed that uptake of both *H37Rv* and *H37Rv/HCL2* was similar by THP-1 cells. However, a drastic reduction of bacterial load after 72 hours was noticed in the mutant HCL2 strain. The control strains such as *H37Rv/pVV16, H37Rv/GFP* and *ΔRD1* continued to grow as reported previously (Figure 3C). Similar reductions in CFU counts were observed when HCL2 was added exogenously over infected THP-1 cells (Figure 3C). Any possible cytotoxic effects of HCL2 on THP-1 cells were studied by MTT assay at 0, 24, 48 and 72 hours after addition of 15 μg/ml HCL2 peptide. No cytotoxicity was observed (data not shown). These observations indicated to us that HCL2 potentially disrupts virulence. It is important to notice here that the inhibition of intracellular growth is similar to that of the avirulent *ΔRD1* mutant, suggesting that disruption of ESAT-6:CFP10 leads to a similar attenuation outcome.

### *M. tuberculosis H37Rv/HCL2* mounts host protective immune response

**Figure 4:**
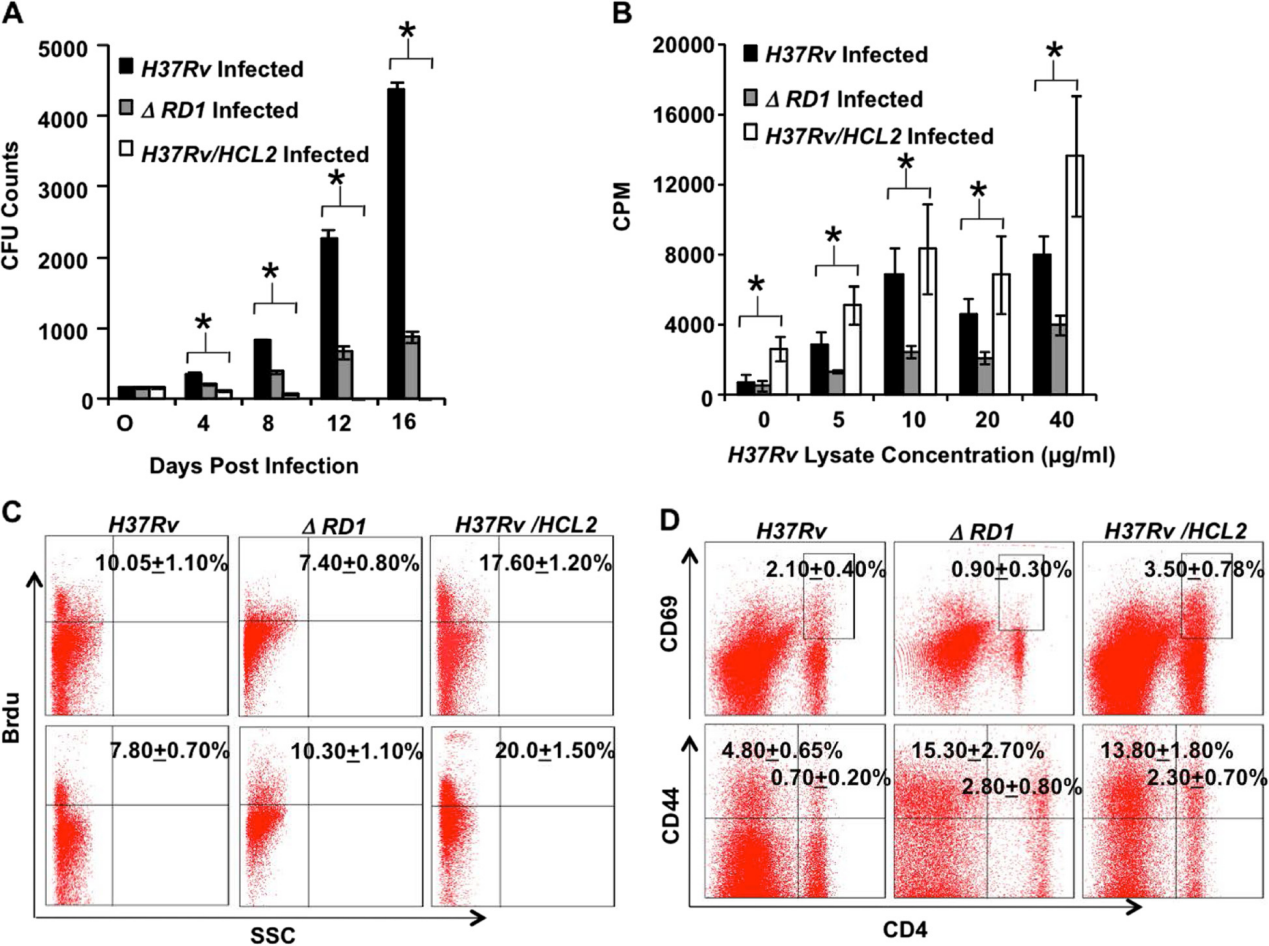
**HCL2 peptide expressed in *M. tuberculosis H37Rv *reduces bacterial burden and activates T-cells. (A)** Bacterial burden in lungs at day 16 in *H37Rv*, *ΔRD1* and *H37Rv/HCL2* infected mice (*p < 0.05). Data shown here is a representative of two independent experiments. Each CFU experiment has been carried out in triplicates (6 mice per experiment). **(B)** Enhanced T cell proliferation in response to *M. tuberculosis* bacterial protein lysate (Complete Soluble Antigen) (*p < 0.05). **(C)** T cell proliferation *in vivo* by Brdu incorporation. Data shown here is representative of three independent experiments with six mice in each group and represents the mean ± STDEV values. **(D)** The percentage of cells expressing CD44 and CD69 among CD4^+^ T cells is shown in the dot-plot with mean ± STDEV. CD44 and CD69 expression by CD4^+^ splenocytes was also found to be significantly higher in *H37Rv/HCL2* infected mice than *H37Rv* infected mice. Data shown here are representative of three independent experiments with six mice in each group.

Our aim was to disrupt virulence but at the same time retain the antigenic property inherent in *H37Rv*. While it is clear that the recombinant strain is indeed prone to faster clearance by the macrophages, for a robust vaccine efficacy, adaptive immunity in the form of both Th1 and Th17 responses is required. Therefore, we tested whether HCL2-expressing *M. tuberculosis* strain can mount Th1 and Th17 responses. We infected BALB/c mice to determine the bacterial burden at different time-points as well as mounting the *M. tuberculosis* antigen-specific immune responses. We observed a significant difference in bacterial burden 8 days post-infection in mice (BALB/c) model between *H37Rv* and *H37Rv/HCL2* strains*.* There was no detectable bacillary load 12 and 16 days post-infection (Figure 4A) in *H37Rv/HCL2* infected mice. Interestingly, we found that clearance of HCL2 recombinant strain is much faster than that of parent *H37Rv* or *ΔRD1* strain. This could either be because a non-functioning ESAT-6:CFP10 complex attenuates this strain*.* This observation indicates that the recombinant strain *H37Rv/HCL2* is avirulent. It has been shown in various studies that strong attenuation of the strain leads to decreased bacterial counts in lungs and spleen [20]. *M. tuberculosis* antigen-specific immune response was determined in infected animals by examining proliferation of splenocytes stimulated with *M. tuberculosis* Complete Soluble Antigen (CSA) 10 days post-infection. A dramatic enhanced antigen-specific hyperproliferatve response was observed in animals infected with *H37Rv/HCL2* as compared to that of *H37Rv* (Figure 4B).

**Figure 5:**
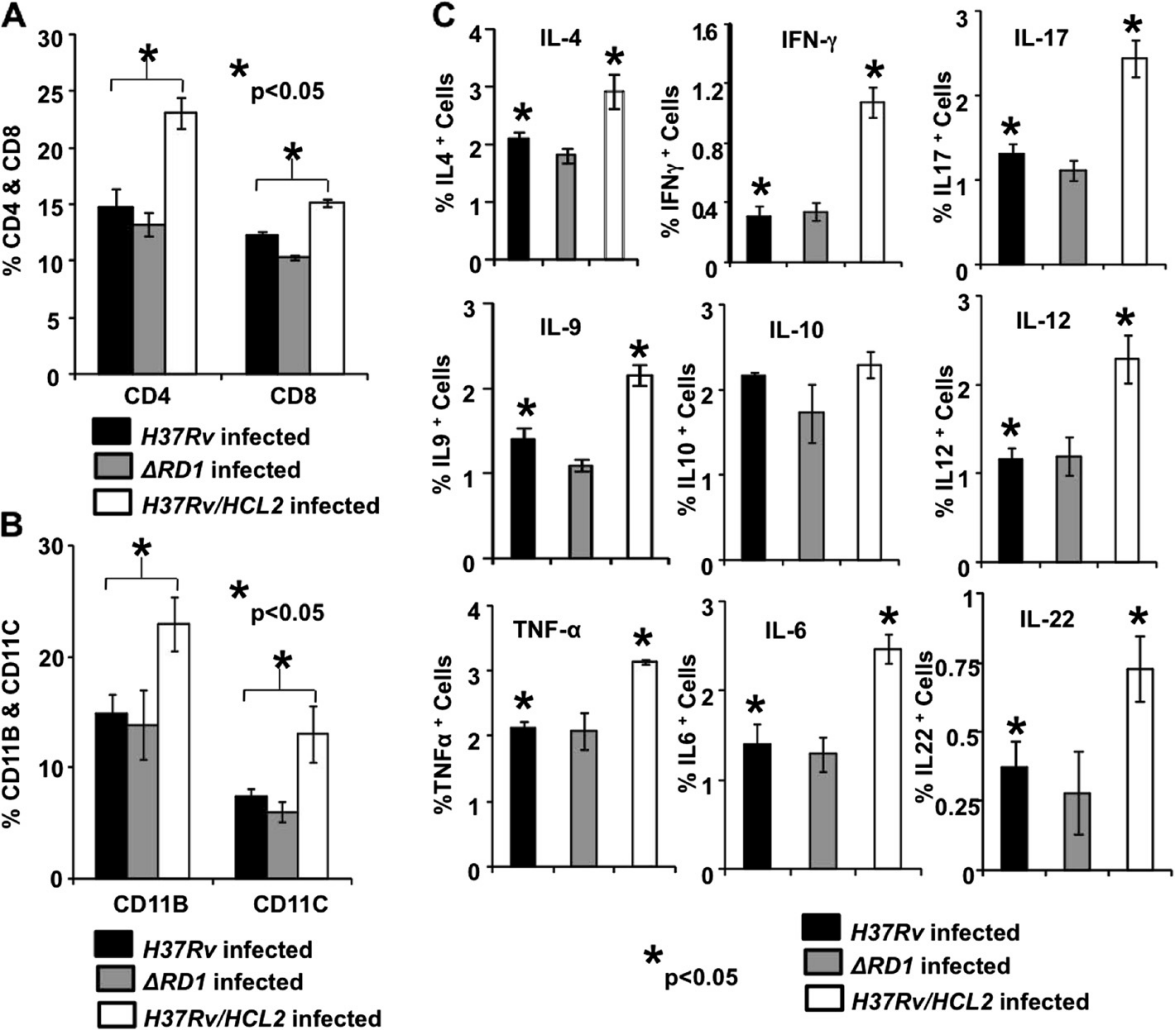
**HCL2 peptide expressed in *M. tuberculosis H37Rv *enhances expression of helper T-cells and prime APCs. (A)** The percentage of splenic T lymphocytes in *H37Rv/HCL2, ΔRD1* and *H37Rv* infected mice expressing CD4 and CD8 is shown in the bar diagram with mean ± STDEV and Student’s T-test (*p < 0.05). **(B)** Increased number of Professional APCs was observed in *H37Rv/HCL2* infected mice as compared to *H37Rv* and *ΔRD1* infected mice at day 10 after infection. The percentage of cells expressing CD11B and CD11C is shown in the bar diagram with mean ± STDEV and Student’s T-test (*p < 0.05). **(C)** Total cytokine production by splenocytes is shown by bar diagram. Data shown with mean ± STDEV and Student’s T-test was applied for estimating significance between two parameters (*P < 0.05).

*In vivo* proliferation of T cells in Splenocytes and Lung T cells was tested by Brdu incorporation and we noticed *H37Rv/HCL2* infected animal had increased T cell proliferation (Figure 4C). We examined CD69 and CD44 expression in CD4^+^ populations and found significantly higher expression (Figure 4D). To study further the immune responses, we focused our attention on CD4, CD8, NK1.1 and Professional APCs. It was observed that CD4, CD8, CD11b and CD11c numbers (Figure 5A & 5B) were dramatically higher compared to the control animals that were infected with *H37Rv*. To check the cytokine milieu in *H37Rv/HCL2* and *H37Rv* infected mice, we isolated the cells from spleen and cultured them with PMA, Ionomycin and *M. tuberculosis* lysate (CSA) in appropriate concentrations overnight. Subsequently, after 6 hrs of BFA treatment, we carried out intracellular staining for IL-4, IFN-γ, IL-17, IL-9, IL-10, IL-12, TNF-α, IL-6 and IL-22. We found no difference in IL-10 but obtained a significant up-regulation of IL-4, IFN-γ, IL-17, IL-9, IL-12, TNF-α, IL-6 and IL-22 (Figure 5C) secretion in *H37Rv/HCL2* infected mice. These data indicated that expression of HCL2 in *H37Rv* attenuates its virulence. However, they retain antigenic property to mount immune response similar to that of virulent strain *H37Rv*. In all experiments, we used *ΔRD1* as a control, which is known to lose its antigenic capacity to mount host protective immune responses. Clearly, our study demonstrated that, although HCL2 recombinant strain was attenuated, it still retained superior antigenic property and mounted a better host immune response than *ΔRD1*.

### HCL2 is not toxic to host cells

**Figure 6:**
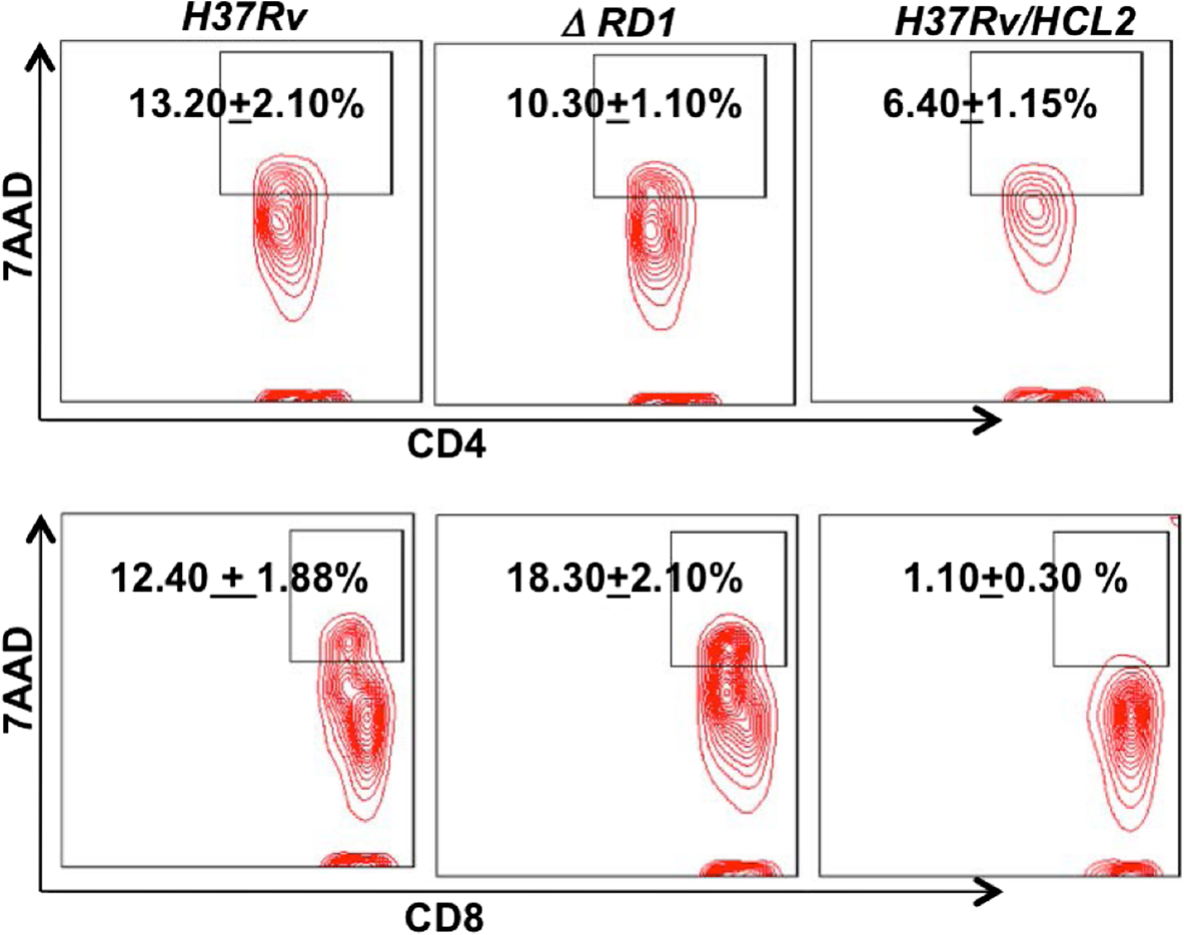
**HCL2 is non-toxic to mice.** Cell death in both CD4^+^ as well as CD8^+^ lung lymphocytes in *H37Rv/HCL2, ΔRD1* and *H37Rv* infected mice. Data showed in Contour plot with mean ± STDEV.

We were intrigued that expression of HCL2 not only attenuates virulence, but also renders slow bacterial growth in liquid culture and disintegrates bacterial cell wall. This raises the question whether HCL2 is itself toxic. To examine this possibility we tested HCL2 on host cells. We did not find any toxicity in mice lung lymphocytes (Figure 6). Furthermore, addition of HCL2 had no effect on the growth of *Escherichia coli* (data not shown). Early apoptotic events were also ruled out on murine macrophages in presence of HCL2 (Additional file 4: Figure S4A). Therefore, toxicity towards bacilli by HCL2 is rather specific.

## Discussion

ESAT-6 is one of the major secretory proteins of *M.tb* and plays a critical role in mycobacterial pathogenesis. In extracellular milieu, it modulates the host immune response and facilitates bacterial spread and dissemination among macrophages and dendritic cells [14,15,21-24]. Furthermore, a large body of literature suggests that effective protection against TB requires both Th1 and Th17 responses from host [3-6]. Role of mycobacterial secretory protein ESAT-6, a protein derived from the region of difference (RD-1), in mounting Th17 responses in the lung has been established earlier by us [3]. The inability of the only known vaccine, BCG, to mount Th17 response can be attributed to the absence of region RD-1 that codes for ESAT-6 and its cognate secretion system. This raises the possibility that RD-1 substituted BCG could serve as a better vaccine. Indeed, immune responses induced by RD-1 recombinant BCG provide superior vaccine efficacy. However, RD-1 substitution in BCG makes the recombinant strain gain virulence, and thus is not suitable for vaccine implication [10,25-27].

We aimed to identify a novel peptide that can incapacitate ESAT-6 and counter its debilitating functions on host system. In our effort to disrupt virulence, we chose to screen peptides from human origin so as to avoid unwanted immune response. The study presented here has demonstrated that HCL2, a peptide from human COX3 protein, strongly binds ESAT-6, disrupts ESAT-6:CFP10 association, and inhibits the growth of *M.tb* in axenic cultures and in macrophages and mice. This is the first example of a peptide disrupting a known protein-protein interaction. HCL2 is part of a seven-bundle helix of COX3 and homology-based modeling shows that it forms a helical structure that interacts with ESAT-6, possibly through hydrophobic interactions – a likely scenario as ESAT-6 itself forms a helix-turn-helix and interacts with the helical arm of CFP10 due to hydrophobic interactions (Additional file 4: Figure S4B).

HCL2 disrupted the strong heterodimeric interaction between ESAT-6 and CFP10 *in vivo* and led to significant reduction in extracellular and intracellular mycobacterial growth. Incapacitation of ESAT-6 by HCL2 could hinder the cell-to-cell spread, as also indicated by effects of HCL2 on intracellular mycobacterium. Therefore, HCL2 has the potential to inhibit *M.tb* spread and pathogenesis inside macrophages.

Interestingly, our electron microscopy data showed distinct degeneration of cell wall and changes in the cell morphology in presence of HCL2 peptide, both endogenous and exogenous. Likewise, mycobacteria expressing HCL2 formed a distinctly smoothened colony. To rule out the general cytotoxic nature of HCL2, its treatment of uninfected THP-1 cell lines and *E. coli* with HCL2 had no effect, suggesting that it is rather specific to *M.tb* (data not shown); in addition *H37Rv/HCL2* infections were non toxic to mice alveolar lymphocytes and peritoneal macrophages.

One possibility is that HCL2 binds and disrupts the secretion system at a location on the bacterial cell surface that harbors the ESAT-6:CFP10 complex, leading to accumulation of toxic material inside the cells resulting in weak growth. This hypothesis is further strengthened by the fact that treatment of *H37Rv* with unrelated peptides had no effect on the growth. Notably, intracellular survival of the pathogen inside human macrophagial THP-1 cell line was also hindered by HCL2. Mycobacterial strain expressing HCL2 was cleared surprisingly fast from the cells and similar effects were observed when cells infected with wild type *H37Rv* were treated with HCL2 exogenously.

To establish the type of immune response raised by HCL2-expressing *H37Rv* (*H37Rv/HCL2*), BALB/c mice were infected with *H37Rv/HCL2* and mycobacterial survival and immune response were studied. The HCL2 expressing strain was cleared in two weeks duration while *H37Rv* continued to grow exponentially showed the normal growth defect. *H37Rv/HCL2* enhanced T cell, macrophage and dendritic cells proliferation and led to activation of CD4^+^ populations. The cytokine milieu in *H37Rv/HCL2* showed significant up-regulation of IFN-γ, IL-17, IL-9, IL-12, TNF-α, IL-6 and IL-22. It is clear that *H37Rv/HCL2* induces host protective immune responses. It was important to find out whether there was a potent enhancement in IL-17, IL6 and IL-22 cytokines, the key regulators of Th17 response, as it is widely believed that Th1 and Th17 responses play important roles in host protective immunity, unlike BCG that is unable to mount a Th17 response. Indeed, we observed that the recombinant strain mounts a strong Th1 and Th17 host protective response, we believe ultimately responsible for the accelerated clearance of this recombinant strain.

This is the first study demonstrating the ability of a novel peptide HCL2 to disrupt the ESAT-6:CFP10 heterodimeric complex and significantly affect growth of both extracellular and intracellular bacteria, as well as cellular morphology and colony texture on solid media. Concomitantly, in preclinical mouse model system, HCL2 has shown significant reduction in mycobacterial infection and strong protective immune response. This study was aimed as a proof-of-concept validation and long-term vaccine efficacy experiments are currently the focus of our laboratories.

## Conclusions

We have demonstrated that disruption of ESAT-6:CFP10 attenuates virulence in *M. tuberculosis* rendering faster clearance in mice. Unlike BCG or *ΔRD1, HCL2* recombinant *M. tuberculosis* retains better host protective immune responses*.* The present study lays the foundation for a possible BCG alternative and thus deserves attention and testing for eventual human application. Further studies, involving direct comparison with BCG, will throw more light on this possibility, while at the same time, separately; HCL2 offers a tantalizing template for the synthesis of anti-TB peptidomimetic molecules.

## Competing interest

The authors declare no financial or commercial conflict of interest.

## Authors’ contributions

Conception and design: AR, GD, SKS, ST. Analysis and interpretation of the data: AR, GD, SKS, ST, DKS, AK, AG, KB, PP. Critical review and revision of the article for scientific accuracy and intellectual content: SKS, ST, DKS, AK, AG, KB, PP, SK, MB, PM, GD, AR. Final approval of the article: SKS, ST, DKS, AK, AG, KB, PP, SK, MB, GD, AR. Statistical expertise: SKS, ST, KB, PP, SK, MB. Collection and assembly of data: SK, ST. All authors read and approved the final manuscript. 

## Pre-publication history

The pre-publication history for this paper can be accessed here:

http://www.biomedcentral.com/1471-2334/14/355/prepub

## Supplementary Material

Additional file 1: Figure S1 Sequences of control peptides ED3-His_6X_ and DL1, used as ‘unrelated peptide controls’ in this study. Sequence of another ESAT-6 binding peptide named SL3 has also been provided (unpublished results). Click here for file

Additional file 2: Figure S2 HCL2 expressing H37Rv/HCL2GFP cells were counted from 8 different fields from different areas and percentage was calculated. Click here for file

Additional file 3 10 fields from different areas were observed by electron microscope and percentage of cell deformations was calculated. Click here for file

Additional file 4: Figure S4 (A) Intraperitoneal macrophages isolated from mice were cultured and, 10 hours post-infection with *H37Rv* and *H37Rv/HCL2* strains in 1:10 ratio, were surface-stained with anti-CD11B, CD11C antibodies followed by Annexin V staining for 40 min followed by flow cytometry to assess pre apoptotic cells. HCL2 expression showed no significant increase in Annexin V/apoptotic cells. The percentage of cells expressing Annexin V among CD11B cells is shown with mean±STDEV. Data shown here are representative of three independent experiments. (B) GRAMM-X server based ESAT-6-HCL2 docking model. Pictorial representation of ESAT-6 interacting with HCL2 as observed through GRAMM-X docking server. (ESAT-6; Red, CFP10; Cyan, HCL2; Blue). Click here for file
