# Supplementary material for: A peptide fragment from the human COX3 protein disrupts association of Mycobacterium tuberculosis virulence proteins ESAT-6 and CFP10, inhibits mycobacterial growth and mounts protective immune response
Source: BMC Infect Dis. 2014 Jul 1;14:355. doi: 10.1186/1471-2334-14-355 (PMC4089558; doi:10.1186/1471-2334-14-355)
Supplement: Additional file 1: Figure S1 — Sequences of control peptides ED3-His6X and DL1, used as ‘unrelated peptide controls’ in this study. Sequence of another ESAT-6 binding peptide named SL3 has also been provided (unpublished results). [file 1471-2334-14-355-S1.pdf]

A Sequence of SL3 (another peptide isolated from lung cDNA library):
peptide strongly binds to ESAT6; unpublished results
AAARIRHEGVFLLIGNSCFSLPRNGPQLLLLAW*

B Sequence of ED3-His$_{6X}$ (Dengue envelop protein domain III):
unrelated protein showed no interaction with purified ESAT6
MGYVGMSYSMCTGKFKVVKEIAETQHGTIVIRVQYEGDGSPCKIPFEIMDLEKRHVLGR
LITVNPIVTEKDSPVNIEAEPPFGDSYIIIGVEPGQLKLNWFKKGSSIGQMIETTMRGAKR
MAILGYVLEHHHHHH*

C Sequence of DL1 (another peptide isolated from lung cDNA library):
unrelated peptide; no significant reduction in *H37Rv* growth both *in vitro* and *ex vivo*
AAARIRHEAGHSMDMRVPAQLLGLLLLWLPGARCAIQLTQSPSSLRASVRGRVTITCRA
SQVMTSFLARFPQKPGKPPEVLIFDASSLERGVPSRFSGSGSGTDFTLTINSLQPDHFAT
DYCQHFSGPSTGDETNCGCTICLHLPAI*

Figure S1
