# Supplementary figures and images for: A peptide fragment from the human COX3 protein disrupts association of Mycobacterium tuberculosis virulence proteins ESAT-6 and CFP10, inhibits mycobacterial growth and mounts protective immune response
Source: BMC Infect Dis. 2014 Jul 1;14:355. doi: 10.1186/1471-2334-14-355 (PMC4089558; doi:10.1186/1471-2334-14-355)

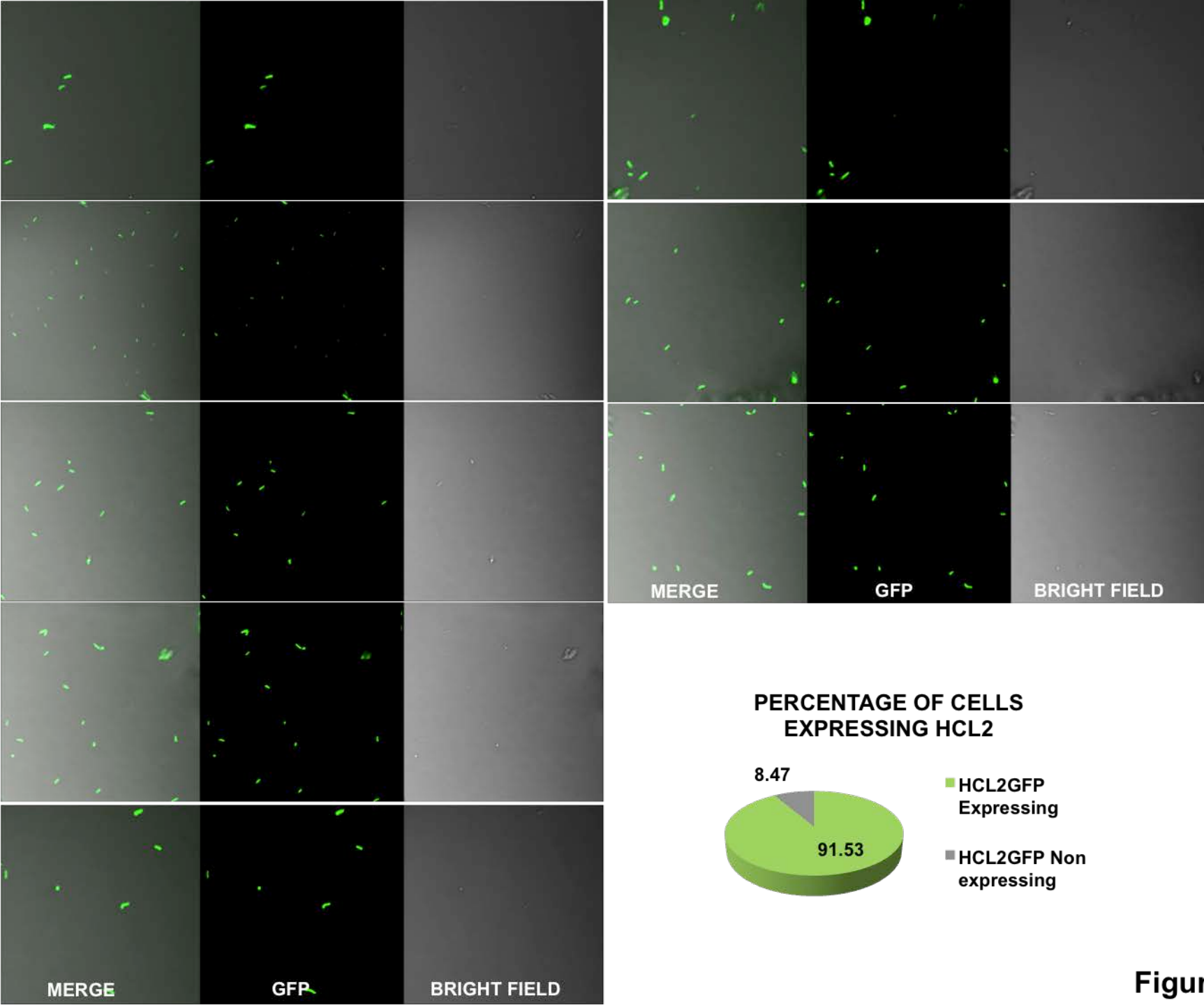

**Figure S2**

Supplement: Additional file 2: Figure S2 — HCL2 expressing H37Rv/HCL2GFP cells were counted from 8 different fields from different areas and percentage was calculated. [file 1471-2334-14-355-S2.pdf]

**H37Rv/pVV16**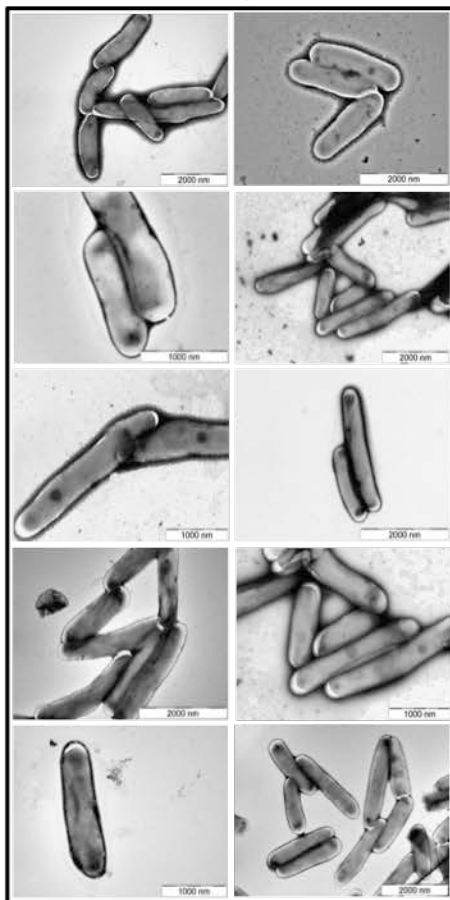**H37Rv/HCL2**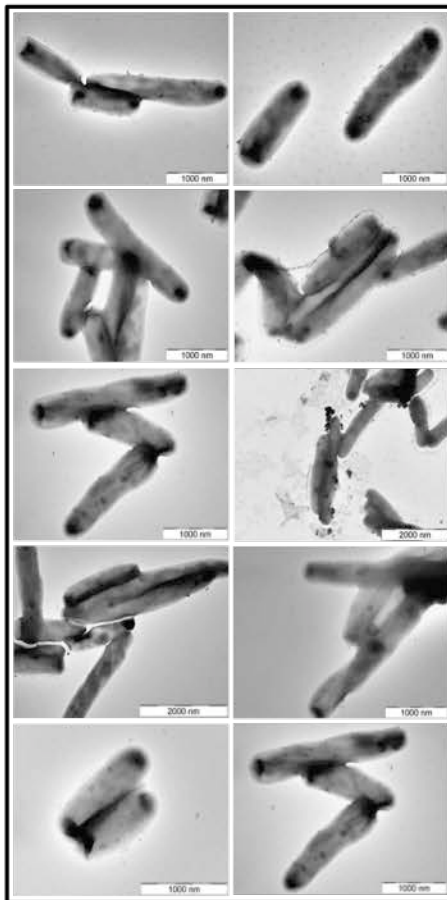**H37Rv**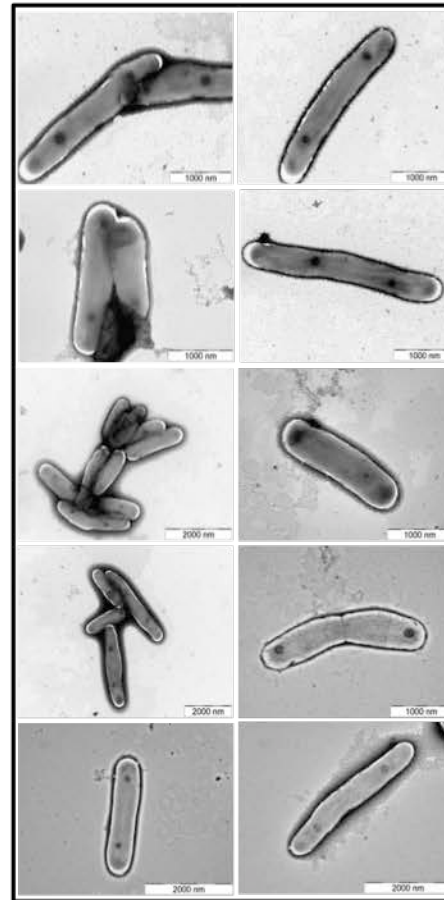**H37Rv+HCL2**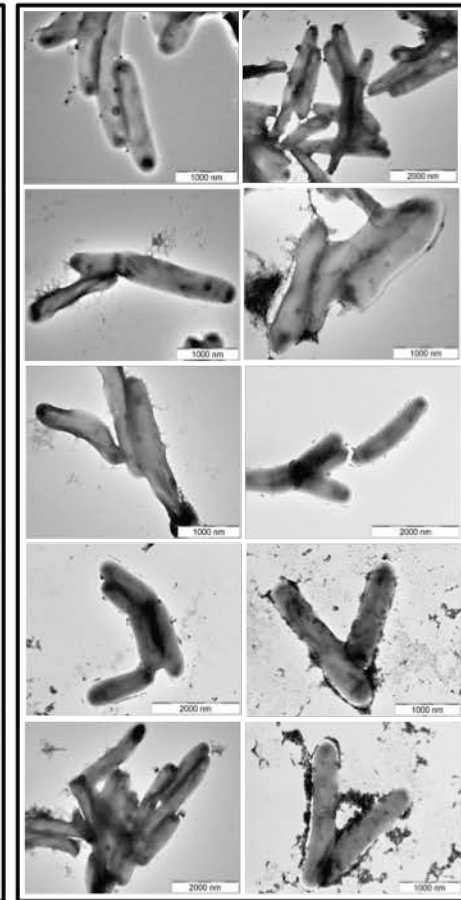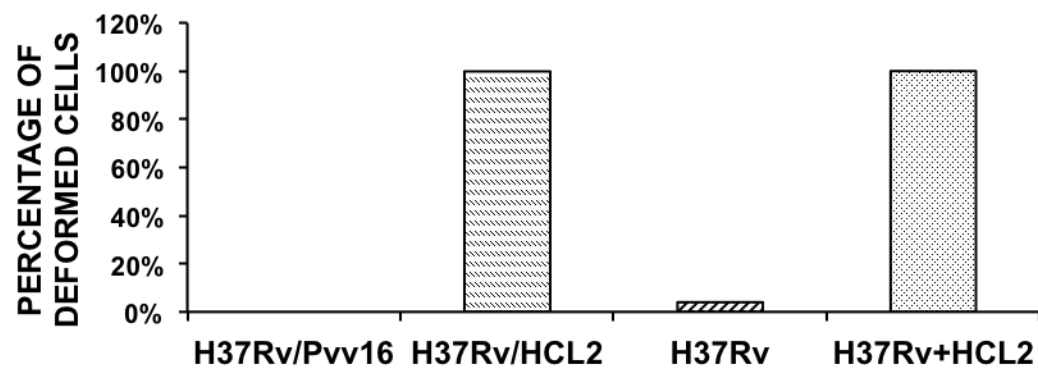**Figure S3**

Supplement: Additional file 3 — 10 fields from different areas were observed by electron microscope and percentage of cell deformations was calculated. [file 1471-2334-14-355-S3.pdf]

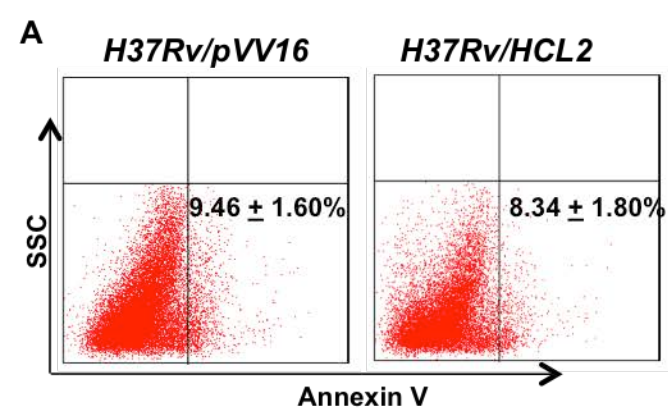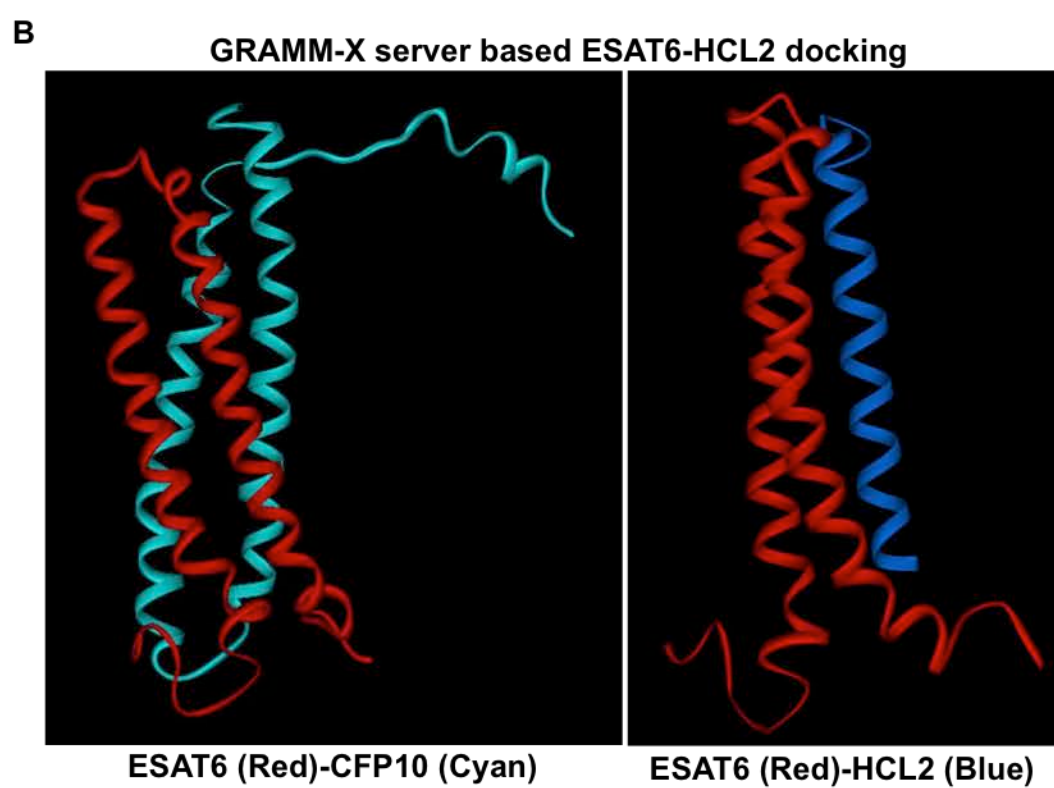

**Figure S4**

Supplement: Additional file 4: Figure S4 — (A) Intraperitoneal macrophages isolated from mice were cultured and, 10 hours post-infection with H37Rv and H37Rv/HCL2 strains in 1:10 ratio, were surface-stained with anti-CD11B, CD11C antibodies followed by Annexin V staining for 40 min followed by flow cytometry to assess pre apoptotic cells. HCL2 expression showed no significant increase in Annexin V/apoptotic cells. The percentage of cells expressing Annexin V among CD11B cells is shown with mean±STDEV. Data shown here are representative of three independent experiments. (B) GRAMM-X server based ESAT-6-HCL2 docking model. Pictorial representation of ESAT-6 interacting with HCL2 as observed through GRAMM-X docking server. (ESAT-6; Red, CFP10; Cyan, HCL2; Blue). [file 1471-2334-14-355-S4.pdf]
